# Primary glomerular disease in adults: pattern over decades,interpretation across the globe

**Published:** 2015-12-01

**Authors:** Rubina Naqvi

**Affiliations:** Sindh Institute of Urology and Transplantation (SIUT), Karachi, Pakistan

**Keywords:** Glomerulonephritis, Immunofluorescence, Renal failure, Membranous nephropathy, Minimal change disease

**Implication for health policy/practice/research/medical education:**

The pattern of glomerular disease in adults varies considerably across the globe and haschanged within same geographical regions and clinical settings. Diagnosis of glomerularlesions on basis of biopsy remains a standard approach.

The pattern of glomerular disease in adults varies considerably across the globe and has changed within same geographical regions and clinical settings. Diagnosis of glomerular lesions on basis of biopsy remains standard despite arguments of margins of error related with sampling in random fashion. Focal segmental glomerulosclerosis (FSGS) which emerged as separate clinic-pathological entity in 1970s has been reported commonest pattern in glomerular pathology (1). The approach to diagnosis of FSGS remained problematic because of morphologic features of its superimposition on other glomerular processes. It remained long standing debate of differentiation of idiopathic with secondary FSGS. (2) Existence of tip lesion in a variety of glomerular disease presenting with proteinuria such as IgA nephropathy, idiopathic membranous nephropathy (IMN) and diabetic nephropathy supports the possibility of this lesion arising through physical stress in setting of severe nephritic syndrome. During the past four decades concepts of FSGS has been refined in more clinic-pathological studies by numerous researchers.

During last four decades IMN reported most common cause of nephritic syndrome in adults worldwide (3). Debate over its management and course of disease continues as long, it is still believed that one third of patients develop spontaneous remission. The second part of controversy is whether we lose ability to treat patients effectively while waiting for spontaneous remission.

IgA nephropathy once reported most frequent cause of glomerulonephritis among patients who undergo renal biopsy. Prevalence of IgA nephropathy to some extent seems to be influenced by policy of proceeding with renal biopsy in all patients coming with microscopic hematuria (4). In our experience we find very little prevalence of IgA nephropathy in our population undergone renal biopsy. We rather see IgM nephropathy more frequently than IgA (1). IgM nephropathy from other centers reported very scarcely.

Minimal change disease (MCD) is less frequent cause of glomerular disease in adults; we have reported 5.8% adults biopsied for nephritic syndrome, acute nephritic syndrome, non-nephritic proteinuria, rapidly progressive glomerular disease and isolated hematuria (1). Study published by Modugumudi et al (5) in current issue of this journal report 17% of adults underwent renal biopsy for similar indications shown MCD. This is a high number found in adults to have MCD as primary glomerular disease. Besides, Modugumudi et al have not performed electron microscopy in these patients which remains hallmark in confirmation of diagnosis of MCD.

Surprisingly FSGS remains very low on list in present study only 6.5% had FSGS as causative pathology. We have reported primary FSGS as most frequent pathological lesion in our studied population which happened to be a large group of 1793 patients. Recently over last 18-20 months we have observed sudden rise in mesangiocapillary pattern in our biopsy population of adult patients (unpublished data).

The present study by Modugumudi et al (5) also claimed to be prospective study, theoretically prospective studies are designed to seek the association of a hypothesized risk or most frequent factor and assess these over a period of time. It would have been more interesting to know if authors follow their hypothesis over studied period and report any deflection from baseline hypothesis and to highlight reasons for such difference.

## Author’s contribution

RN was the single author of the manuscript.

## Conflicts of interest

The author declared no competing interests.

## Ethical considerations

Ethical issues (including plagiarism, data fabrication, double publication) have been completely observed by the author.

## Funding/Support

None.
